# Critique of Well Activity Proxy Uses Inadequate Data and Statistics

**DOI:** 10.3390/ijerph17155597

**Published:** 2020-08-03

**Authors:** Gunnar W. Schade

**Affiliations:** Department of Atmospheric Sciences, Texas A&M University, College Station, TX 77840-3150, USA; gws@geos.tamu.edu

**Keywords:** well activity metric, air pollutants, unconventional natural gas development, Kappa statistic

## 1. Introduction

The recent publication, “Assessing Agreement in Exposure Classification between Proximity-Based Metrics and Air Monitoring Data in Epidemiology Studies of Unconventional Resource Development” by Hess et al. claims to perform a validation of well-activity (WA) proximity models used in epidemiologic research studies of unconventional natural gas development (UNGD) [1]. While a previous comment already outlined several perceived flaws of this work [2], here I focus on and question both the premises and the conclusions of their work, based upon the selected air pollutants and the selected statistical test, respectively. The results presented in their work are inadequate to claim that “potential exposure misclassification can be assessed” through “general agreement between exposure classifications based on WA and air pollutant concentrations” [3]. I use the existing scientific literature to question the premises, and a simple model to demonstrate the inadequacy of the statistical test results presented.

## 2. Inadequacy of Selected Air Pollutant Data

The authors selected a set of air pollutants and monitor locations for their study that appear to have been made solely by the convenience of their existence, not based on any direct past claims of this particular subset of air pollutants as contributed to by UNGD and as making significant contributions to observed public health effects. The authors themselves lay out in their Introduction that most of these air pollutants are not strongly affected by UNGD emissions, but their review of the air quality literature is selective and not representative. As explained in review articles, such as Allen [4] or Costa et al. [5], the dominant emissions from UNGD are hydrocarbons (HCs), a subset of VOCs, that are indirectly reflected in Hess et al.’s listing of the industry’s contributions to the Pennsylvania emission inventory. Criteria air pollutants, such as CO, PM, NO_x_, and ozone, are not emitted in amounts affecting air quality at monitoring stations significantly; nor do they affect air quality dominantly as a result of flaring and truck traffic [6], respectively. Ozone is a special case, as it is a secondary air pollutant strongly affected by NO_x_ and HC emissions. Its formation can be boosted regionally by oil and gas development (e.g., in [7]), and this may affect legal NAAQS evaluations in nearby populated areas that monitor ozone.

Instead of using this information to carefully select input data, Hess et al. [1] selected two studies from Pennsylvania only, reasoning that ambient pollutant levels, specifically those air pollutants they selected, are most likely not significantly affected by UNGD. Thereby, they arguably predetermined the outcome of their own study. Furthermore, while their first choice was a limited study focused on UNGD site emissions, not their effects on regional concentrations, the second, a report by the Pennsylvania DEP, only carried out a limited analysis of its own monitoring efforts. Rather than constraining information to these limited studies, to determine the relative impacts of UNGD activities on air quality, source apportionment analyses are needed (e.g., in [8]), which can describe the contributions to ambient air quality of a particular source relative to other sources while giving the sources’ chemical fingerprints. As such, these results would have provided a more appropriate air pollutant screen to test against the well activity (WA) metric.

## 3. Inadequacy of Selected Statistical Method

While the selected air pollutant data were not likely to be goal-oriented with respect to the authors’ stated aims, it stands to reason that even a highly limited contribution of UNGD to the selected air pollutants could be associated with certain public health effects. Although the WA metric does not claim to represent air quality effects only [2], Hess et al. argue that it could serve as a proxy of UNGD-related exposure at air quality monitoring sites. Assuming that this is correct, then regardless of the strength of the association between air pollutant and emissions proxy, a statistical test could reveal the possible significance. Hess et al. selected an inter-rater reliability test, called a Kappa-statistic, to test for a possible association by dividing both the air quality data and the WA metric data into quartiles. Their testing found no “agreement” between the four “exposure categories” and the four WA metric categories. While it has previously been pointed out that the Kappa-statistic is likely not an appropriate statistic for the data at hand [2], the authors argue in their reply that its measure of “general agreement between exposure classifications based on WA and air pollutant concentrations” can be used to assess “potential exposure misclassification” [3]. They do not offer an example though, and a similar use cannot be found in the literature. Here, I offer a simple test to assess whether the Kappa test, as applied by Hess et al., can indeed be used to assess “agreement” between two continuous data sets. I used *R* software [9] to create a data set of 1000 random values (akin to approximately three years of continuous daily data) from a log-normal distribution (air quality data are typically log-normally distributed [10]). Next, I consecutively added an increasing amount of white noise to the original data set while keeping track of: (i) the correlation between the original and its “noisy” self; (ii) the correlation between the original data and its noisy self—both arranged into quartiles; (iii) the Kappa statistic (using function *kappa2* in *R*-package *irr*) between those quartiles. The results are shown in Table 1 (The commented R-code used is shown in Supplementary Materials). While the correlation between the data sets keeps degrading, as expected, the significance of the correlation (*p*-value) is maintained to a very high noise level, despite the determination coefficient suggesting that the relationship is so weak as to explain five or fewer percent of the data set’s variance. Once the data sets are aggregated into quartiles, meaning transformed to ordinal categories, the correlation (r^2^ value) naturally degrades somewhat, but its high statistical significance is nevertheless maintained, except for the highest noise cases. The same, however, is not the case for the Kappa statistic, which degrades into the arbitrary “none to poor” range already at comparatively small noise levels. Curiously, Hess et al. did not report on the Kappa statistic’s significance in their setting. Notably, for the deliberately correlated data in this case study, Kappa remains statistically significant at the 95% level to comparatively high noise levels despite the actual Kappa degrading to less than 0.05 in some comparisons. These results did not fundamentally change when using normally instead of log-normally-distributed data (not shown). Since we know the data sets are associated, this shows that the Kappa statistic is not capable of revealing this relationship unless its arbitrary “strength of agreement” is at least amended with a statistical significance criterion, and even then it does not capture the underlying correlation between the continuous data sets adequately. Since it does so by the nature of its comparison, it can be characterized as inadequate to deliver the authors’ aim, as it cannot reveal a weak relationship for the data at hand even when we know one is present.

## 4. Conclusions

Recent findings of significant associations between a well activity metric—used as a proxy for environmental impacts—and public health data have led to increased awareness of the potential risks of the renewed US oil and gas boom. While the WA metric is arguably a poor proxy for environmental impacts, given the sparsity of environmental measurements, including air quality monitoring in relevant areas, it currently serves to highlight potential associations. These associations are informed by knowledge about the compounds and toxicity of emissions from the oil and gas industry, such as, for example, endocrine disrupting compounds. This suggests that more detailed exposure studies may be necessary and the industry could fill some of these gaps by providing insight into emissions and exposure pathways, or by providing air quality measurements in shale production areas. Instead, the authors presented a poorly conceived study that claims to demonstrate a major weakness of the WA metric, but does so using inadequate premises and inadequate analyses.

## Figures and Tables

**Table 1:** Results from statistical tests on log-normally distributed data (*n* = 1000), comparing determination coefficients (r^2^ = “rsq”) and p-values of raw data with those of quartile-transformed data and the Kappa-statistic of those data. Columns represent the white noise level added to the raw data to create a correlated data set to be compared with the original. In this case, the third column represents a white noise level slightly larger than the SD of the data, while the last column (20) represents a noise level approximately ten-times one SD.

**Statistic**	**1**	**2**	**3**	**4**	**5**	**6**	**7**	**8**	**9**	**10**
**raw_rsq**	0.937	0.6961	0.5882	0.2973	0.3169	0.3281	0.2131	0.1287	0.1193	0.1636
**raw_p**	0	0	0	0	0	0	0	0	0	0
**quart_rsq**	0.596	0.3109	0.1697	0.0944	0.0949	0.0788	0.0502	0.041	0.0369	0.0248
**quart_p**	0	0	0	0	0	0	0	0	0	0
**kappa**	0.428	0.1973	0.14	0.1227	0.0987	0.0867	0.0707	0.0733	0.068	0.056
**kappa_p**	0	0	0	0	0	0	0.0001	0.0001	0.0002	0.0022
	**11**	**12**	**13**	**14**	**15**	**16**	**17**	**18**	**19**	**20**
**raw_rsq**	0.1469	0.0656	0.0724	0.0354	0.06	0.0444	0.0503	0.0455	0.0202	0.0461
**raw_p**	0	0	0	0	0	0	0	0	0	0
**quart_rsq**	0.0282	0.0164	0.0274	0.0189	0.0221	0.0082	0.0108	0.0085	0.0072	0.0236
**quart_p**	0	0	0	0	0	0.0042	0.001	0.0036	0.0073	0
**kappa**	0.0373	0.056	0.0613	0.048	0.0587	0.028	0.0507	0.04	0.024	0.056
**kappa_p**	0.0409	0.0022	0.0008	0.0086	0.0013	0.1251	0.0055	0.0285	0.1887	0.0022

## Supplementary Materials

The following are available online at https://www.mdpi.com/1660-4601/17/15/5597/s1: Commented R-code used to generate Table 1.
